# Alternation in functional connectivity within default mode network after psychodynamic psychotherapy in borderline personality disorder

**DOI:** 10.1186/s12991-023-00449-y

**Published:** 2023-05-11

**Authors:** Saba Amiri, Fatemeh Sadat Mirfazeli, Jordan Grafman, Homa Mohammadsadeghi, Mehrdad Eftekhar, Nazila Karimzad, Maryam Mohebbi, Shabnam Nohesara

**Affiliations:** 1 Neuroscience Research Center, Shahid Beheshti University of Medical Science, Tehran, Iran; 2 Department of Psychiatry, School of Medicine, Mental Health Research Center, Psychosocial Health Research Institute, Iran University of Medical Sciences (IUMS), Tehran, Iran; 3 Department of Physical Medicine & Rehabilitation, Neurology, Cognitive Neurology and Alzheimer’s Center, Department of Psychiatry, Feinberg School of Medicine & Department of Psychology, Weinberg College of Arts and Sciences, Northwestern University, Chicago, IL USA; 4 Iran Psychiatric Hospital, Iran University of Medical Sciences (IUMS), Tehran, Iran; 5 Islamic Azad University Science and Research Branch Qazvin, Qazvin, Iran

**Keywords:** Default mode network, Connectivity, Resting-state fMRI, Borderline personality disorder

## Abstract

**Background:**

Borderline personality disorder (BPD) is characterized by impairments in emotion regulation, impulse control, and interpersonal and social functioning along with a deficit in emotional awareness and empathy. In this study, we investigated whether functional connectivity (FC) within the default mode network (DMN) is affected by 1-year psychodynamic psychotherapy in patients with BPD.

**Methods:**

Nine BPD patients filled out the demography, Interpersonal Reactive Index (IRI), Toronto Alexithymia Scale 20 (TAS 20), the Alcohol, Smoking, and Substance Involvement Screening Test (ASSIST), and the Borderline Evaluation Severity over Time (BEST) questionnaire. The BPD group (9F) and the control group (9F) had a mean ± SD age of 28.2 ± 5.3 years and 30.4 ± 6.1 years, respectively. BPD subjects underwent longitudinal resting-state fMRI before psychodynamic psychotherapy and then every 4 months for a year after initiating psychotherapy. FC in DMN was characterized by calculating the nodal degree, a measure of centrality in the graph theory.

**Results:**

The results indicated that patients with BPD present with aberrant DMN connectivity compared to healthy controls. Over a year of psychotherapy, the patients with BPD showed both FC changes (decreasing nodal degree in the dorsal anterior cingulate cortex and increasing in other cingulate cortex regions) and behavioral improvement in their symptoms and substance use. There was also a significant positive association between the decreased nodal degree in regions of the dorsal cingulate cortex and a decrease in the score of the TAS-20 indicating difficulty in identifying feelings after psychotherapy.

**Conclusion:**

In BPD, there is altered FC within the DMN and disruption in self-processing and emotion regulation. Psychotherapy may modify the DMN connectivity and that modification is associated with positive changes in BPD emotional symptoms.

## Highlights

- BPD presented with aberrant DMN connectivity compared to a matched HC group.
- After 1 year of psychotherapy BPD showed behavioral improvement in their symptoms.
- After psychotherapy BPD showed hypo-connectivity in dACC along with an increase in emotional awareness.

## Background

Borderline personality disorder (BPD) is a severe mental illness with a relatively high prevalence among the population, 1% and 10 to 12% in the clinical outpatient setting [1]. It is characterized by impairments in emotion regulation, impulse control, and interpersonal and social functioning [2]. They may also have difficulty in comprehending their own feelings also known as alexithymia [3]. People with BPDs also show deficits in mentalizing [4] and self-awareness [5], two processes that give us the capacity to understand our inner world. Furthermore, individuals with BPD do not merely have problems understanding their own emotions but also understanding and communicating others’ emotions, i.e. empathizing [6].

In patients with BPD with disturbed self-image, identity, and empathizing [6], resting-state functional connectivity is abnormal [7]. Interaction of default mode network (DMN) shapes our sense of self [8, 9]. DMN is involved in self-related mental activity [10]. Its connectivity is also associated with emotional awareness [9]. The DMN is a series of brain areas that normally de-activate during task performance that needs external attention. It includes two midline areas, one located anteriorly in the medial prefrontal cortex (mPFC) and it contains medial prefrontal cortex (mPFC), dorsal medial prefrontal cortex (dmPFC), anterior cingulate cortex(ACC), posterior cingulate cortex (PCC), anterior temporal lobe, inferior frontal gyrus and lateral parietal cortex and one posteriorly in the posterior cingulate cortex/precuneus, and it contains PCC, posterior inferior parietal lobule, angular, hippocampal and temporal lobe [11–13]. Anterior DMN seems to recruit more when reflecting on the present while posterior DMN seems to recruit more when reflecting on the future [14, 15]. In the study of Wolf [7], patients with BPD had decreased functional connectivity of the left inferior parietal lobule and the mid-left temporal cortex in the DMN. In another study patients with BPD showed increased medial prefrontal cortex and right precuneus/posterior cingulate cortex activity in PET scans [16]. The prognosis of BPD with these neurobiological changes over time is variable. Externalizing symptoms like self-destructive behaviors, impulsive reactions, and aggression tend to decline over time [17, 18] while internalizing symptoms such as identity confusion and sense of emptiness, which are the primary sources of suffering in these patients, may persist throughout life [19]. As a consequence, patients with BPD are high-utilizers of medical resources. There are no FDA-approved medications for BPD and research in this field is more limited than other psychiatric conditions with similar or even fewer morbidities [20, 21].

Given the lack of therapeutic medications, the leading treatment choice is psychotherapy. There are various psychodynamic approaches for patients with BPD; as many as eight different therapies for the treatment of BPD have been demonstrated to be effective in randomized controlled trials [22]. The primary mechanism of change in all psychotherapeutic interventions is improving patients’ communication with the external world [23]. Despite the modest efficacy of psychotherapeutic interventions, there is minimal evidence whether a baseline evaluation of BPD can predict patient response to psychotherapeutic or medical interventions [24, 25]. In addition, research on the impact of BPD psychodynamic psychotherapy on DMN functioning, empathetic behavior, and emotional awareness is lacking [6, 26].

Below we report on DMN functional connectivity patterns in patients with BPD compared to healthy control and their alteration after 1 year of psychodynamic psychotherapy. We also explored the association of this alteration with improvements in clinical symptoms, emotional awareness, and empathy. We hypothesized that psychotherapy would improve DMN dysfunction, and that improvement would be associated with improvement in emotional self-awareness (decrease in alexithymia) and emotional communication (empathy).

## Methods and materials

### Participants

Thirteen patients with BPD referring to clinics and psychiatric wards of Iran University of Medical Sciences were recruited. The diagnosis was based on a Structured Clinical Interview for Diagnostic-II (SCID-II) by trained examiners. Patients younger than 18 years old, or older than 50 years old were excluded as were patients with a major neurological disorder such as epilepsy, traumatic brain injury, comorbidity with antisocial personality disorder, substance use disorder during the year of study, alcohol or cannabis intoxication, major mood disorder, psychotic disorder, education lower than high school. To be enrolled in the study, patients were required to be stable on their medications for at least 1 month before recruiting. Participants entered the study after they were fully informed about the 1-year duration and the research purpose and completed an informed consent. Four participants dropped out during 1 year of psychotherapy and they were excluded from the analyses. The BPD group (9F) and the control group (9F) had a mean ± SD age of 28.2 ± 5.3 (range: 20–34) years and 30.4 ± 6.1 (range: 24–35) years, respectively. All the research was approved by the ethical committee of the Iran University of Medical Sciences (Ethical code: IR.IUMS.REC.1398.872) and informed consent was taken from all the participants. All methods were performed in accordance with the relevant guidelines and regulations of Helsinki.

### Instruments

#### Structured Clinical Interview for Diagnostic I (SCID I)

This is a semi-structured interview for examination of major axis I psychiatric disorders based on DSM IV/DSM IV-TR criteria. It usually starts with an open-ended question:” have you ever had “and it is followed by multiple questions about the content. The Kappa values ranged from 0.61 to 0.83, with a mean Kappa of 0 [27]. Its reliability and validity in the Persian translation has been established and its test–retest reliability is fair to good [28–32].

#### Structured Clinical Interview for Diagnostic II (SCID II)

The Structured Clinical Interview for Diagnostic II (SCID-II), carried out as a semi-clinical structured interview, is conducted to diagnose personality disorders based on the DSM IV/DSM IV-TR. Like SCID I, it starts with an open-ended question:” have you ever had “and it is followed by multiple questions about the content. The SCID-II shows adequate interrater reliability (from 0.48 to 0.98) and good reliability for dimensional diagnosis (from 0.90 to 0.98) and internal consistency (0.71–0.98). The SCID-II questionnaire was translated into Persian but its psychometric investigation is somewhat limited. In one study, the Persian SCID II test–retest reliability was reported at 0.87 [33].

#### The Alcohol, Smoking, and Substance Involvement Screening Test (ASSIST)

This scale was first developed to evaluate a wide range of substance use and consequent problems in primary care patients. The ASSIST items were considered easy to answer and were found to be reliable and feasible to administer in an international study. The test–retest reliability coefficients ranged from 0.58 to 0.9. The reliability range for different categories of substances averaged 0.61 for sedatives to 0.78 for opioids [34, 35].

#### Borderline Evaluation of Severity Over Time Questionnaire (BEST)

This is a self-report measure that assesses the change and severity of BPD, such as thoughts, feelings, and negative actions over time. It includes 15 items and three subscales on the Likert-like Range [36]. An example of scale A (thoughts and feelings) would be one should rate how much “feeling angry” causes distress or problems for him/her. It has been suggested as both a reliable (Cronbach’s alfa: 0.86) and valid instrument for change and severity of BPD [36]. Its reliability and validity have been studied in Persian [37].

#### Interpersonal Reactive Index (IRI)

The IRI is a self-report questionnaire. It assesses four dimensions of empathy, and each subscale (empathic concern, perspective-taking, personal distress, and fantasy) is made up of 7 items. An example of empathic concern would be: “*I often have tender, concerned feelings for people less fortunate than me.*” Participants rate how much an item will describe them on a 5-point Likert scale. (Does not describe me well = 0 to represent me very well = 4). The maximum and minimum total scores on this questionnaire are 28 and 0, respectively. This questionnaire has shown relatively good psychometrics with high internal consistency (Alfa Cronbach of 0.71 to 0.77 [38–40] Test–retest reliability was reported within 0.62 to 0.71 [38–40]. This questionnaire has been translated into Persian and its psychometric properties have been studied extensively [41].

#### Toronto Alexithymia Scale-20 (TAS-20)

This questionnaire evaluates four dimensions of emotional awareness, including difficulty identifying the feeling, difficulty describing feelings, and externally oriented thinking. An example would be to rate how much one agrees (from strongly disagree to strongly agree) with every item like “*I am often confused about what emotion I am feeling*”. Validity and reliability have been studied by Bagby et al. [42] and has shown fairly good reliability (Cronbach’s alfa from 0.8 to 0.83) and validity in Persian [43–45].

### Psychotherapy

Once weekly, patients had a session of therapeutic session emphasizing the Transference Focused Psychotherapy approach. The content of the session was written by the therapist every week. The patient's progression through the year was noted and analyzed. Core concepts of transference and countertransference, defense mechanisms, and signs of emotional communication were highlighted. Our focus was based on the way participants relate to the objects and the cue to the object relationships would come from the pattern of transference communication and competition repetitions in the sessions. Each therapist had an individual supervisor. In addition, monthly group supervisory sessions were held to work through the dynamics of the sessions and feelings of being part of a study.

### Procedure

Participants were recruited by convenience sampling among patients referred to clinics and psychiatric wards of the Iran University of Medical Sciences. Those who meet inclusion criteria based on the SCID II interview entered the study. After completing the informed consent, participants with BPD filled out the demographic questionnaire, IRI, TAS 20, ASSIST, BEST questionnaire. Then their default mode network connectivity was measured using resting-state fMRI before initiating psychodynamic psychotherapy. After starting psychodynamic psychotherapy, DMN connections were assessed 3 more times with each assessment separated by approximately 4 months (so that including their baseline assessment, the total number of fMRI assessments for each individual was 4). Finally, at the conclusion of their therapy patients again completed the IRI, TAS 20, ASSIST, the BEST questionnaire to monitor any possible changes.

### fMRI acquisition

Multimodal MRI data were collected in a Siemens magnetom Prisma 3T MRI scanner. The resting-state functional MRI images covering the whole brain were obtained with an echo-planar imaging sequence with the following parameters: 240 volumes (8 min and 6 s), axial slices = 32 slices with 3.5 mm thickness, repetition time (TR) = 2000 ms, echo time (TE) = 30 ms, flip angle (FA) = 90°, voxel size: 3.1 × 3.1 × 3.5 mm, the field of view = 200 × 200 mm, and matrix size = of 64 × 64. T1-weighted structural images were acquired for co-registration of functional images using a sagittal 3D-magnetization prepared rapid acquisition gradient echo (MPRAGE) sequence: TR 1800 ms, TE = 3.53 ms, inversion time (TI) = 1100 ms, FA = 7°, FOV = 256 × 256 mm^2^, matrix size = 256 × 256, slice thickness = 1 mm, and scan time = 4 min and 12 s.

### fMRI analysis

The analyses consist of five sequential steps that included pre-processing, extracting DMN FC matrix (FCM) based on the automated anatomical labeling (AAL) atlas, thresholding, and binary FCM, constructing binary graph network from binary FCM and extracting graph-theoretical features, and finally comparison and statistical analyses.

#### Preprocessing

For each subject, preprocessing of the rs-fMRI data was carried out using statistical parametric mapping (SPM12) and the data processing assistant for resting-state fMRI (DPARSF) toolbox version 4.5 [46]. Briefly, the following steps were carried out: (1) removing the first 10 volumes of the 240 volumes to allow for magnetization equilibrium; (2) skull stripping was performed on both functional and structural images to remove non-brain tissue before co-registration of T1 images and functional images for better registration of T1 image to functional space; (3) slice-timing correction; (4) correcting for head movements, which required the images to be realigned with a six-parameter (rigid body) linear transformation. Individual structural images were co-registered to mean functional images; (5) segmentation of T1-weighted images into grey matter (GM), white matter (WM), and cerebrospinal fluid (CSF); (6) regressing out of 27 nuisance covariates, including signals from WM and, CSF, global signals, and Friston 24 motion parameters; (7) spatial normalization was done to the standard template Montreal Neurological Institute (MNI) space; (8) spatial smoothening with a Gaussian kernel of 6 mm full-width at half-maximum (FWHM_); and (9) subsequently, a temporal band pass filter (0.01–0.01 Hz) was performed to reduce the influence of low-frequency drift and high frequency respiratory and cardiac noise.

#### Brain functional connectivity matrix (FCM) and graph construction

For analysis of FC, the seed regions of the default mode network (DMN) were chosen based on a priori knowledge [47, 48] from the AAL atlas [49] with the DMN regions shown in Table 1. The time series for each region were extracted and then on each pair, the Pearson correlation was used to obtain a correlation matrix for each participant. Based on the correlation matrices, we constructed a weighted brain graph or weighted functional connectivity matrix using a set of sparsity thresholds ranging from 5 to 40% with a step of 1% (5 ≤ *T* ≤ 40). The sparsity threshold represented the proportion of the present connections to the maximum possible connections within the network. This approach included assigning labels 1 to D% (density) of the strongest connections in each network and 0 to other connections [50]. For group comparison, unlike the absolute threshold, the use of proportional thresholds ensures that the network of each group have the same number of nodes and edges [50]. This makes more meaningful comparisons between the two groups. We described FC with a network density of 5–40%. The range of 5–40% was chosen for interpretation, because, according to previous reports, this range is in overall consistency with the biological background of the brain functional networks [51, 52].

**Table 1:** Regions of interest default mode network (DMN)

DMN regions (or nodes)	Abbreviations
Left and right superior frontal gyrus, orbital part	ORBsub.L, ORBsub.R
Left and right middle frontal gyrus	MFG.L, MFG.R
Left and right inferior frontal gyrus, orbital part	ORBinf.L, ORBinf.R
Left and right superior frontal gyrus, medial	SFGmed.L, SFGmed.R
Left and right superior orbital frontal gyrus, medial	ORBsupmed.L, ORBsupmed.R
Left and right anterior cingulate and paracingulate gyri	ACG.L, ACG.R
Left and right median cingulate and paracingulate gyri	DCG.L, DCG.R
Left and right posterior cingulate gyrus	PCG.L, PCG.R
Left and right hippocampus	HIP.L, HIP.R
Left and right parahippocampal gyrus	PHG.L, PHG.R
Left and right inferior parietal lobule	IPL.L, IPL.R
Left and right angular gyrus	ANG.L, ANG.R
Left and right precuneus	PCUN.L, PCUN.R
Left and right middle temporal gyrus	MTG.L, MTG.R
Left and right temporal gyrus pole: middle temporal	TPOmid.L, TPOmid.R
Left and right inferior temporal gyrus	ITG.L, ITG.R

#### Graph-theoretical measures

Centrality metrics can determine the importance of each node in a brain network, which makes them appropriate measures to capture the complexity of functional connectivity. Among these metrics, the nodal degree is the most popular measure of centrality since it is directly related to functional connectivity [53–55]. Furthermore, the nodal degree is shown to have a high correlation with other centrality metrics (betweenness centrality, clustering coefficient, node neighbor’s degree, and closeness centrality) [53, 56–58]. We calculated the ‘*nodal degree*’ in DMN regions and compared patients with BPD with the healthy control group.

The ‘*nodal degree*’ of each node equals the total number of edges that are connected to a node [54].

$$\left( D \right)_{i} = \sum\nolimits_{j \in N} {a_{ij} } ,$$

where *N* is the number of all nodes in the network, *a*_*ij*_ is the connection value between a pair of nodes (*i* and *j*), with *a*_*ij*_ = 1 when a connection between (*i*, *j*) exists, and *a*_*ij*_ = 0 unless otherwise.

Given that nodal degree quantifies FC, it can be concluded that a brain region with a greater/lesser nodal degree has a greater/lesser FC (hyper- and hypoconnectivity). Hyperconnectivity means increased nodal degree in a network and hypoconnectivity means decreased nodal degree in a network in this literature [55].

#### Statistical analyses

A nonparametric permutation test with 10,000 resamples was used to evaluate the significance of differences in *degree* between the HC and BPD groups. The nonparametric permutation test is used to determine whether a measured effect is genuine or is a statistical anomaly due to the randomness associated with the selection of the sample [59]. Permutation testing for controlling the nominal type I error is considered acceptable [59]. We also used a non-parametric permutation test to assess the significance of the differences between groups (reported as *P* values) and to determine the 95% confidence intervals [60].

To determine the relationship between nodal degree results and clinical variables, Pearson correlation coefficients were calculated using SPSS 25 (SPSS Inc., Chicago, Illinois). The clinical variables included the empathy, ASSIST, TAS, and BEST measures. Also, we used paired *t* tests to evaluate differences between pre-and post-psychotherapy clinical evaluations.

## Results

### DMN alternation in BPD during psychotherapy compared to the HC

#### Baseline

**Fig. 1:**
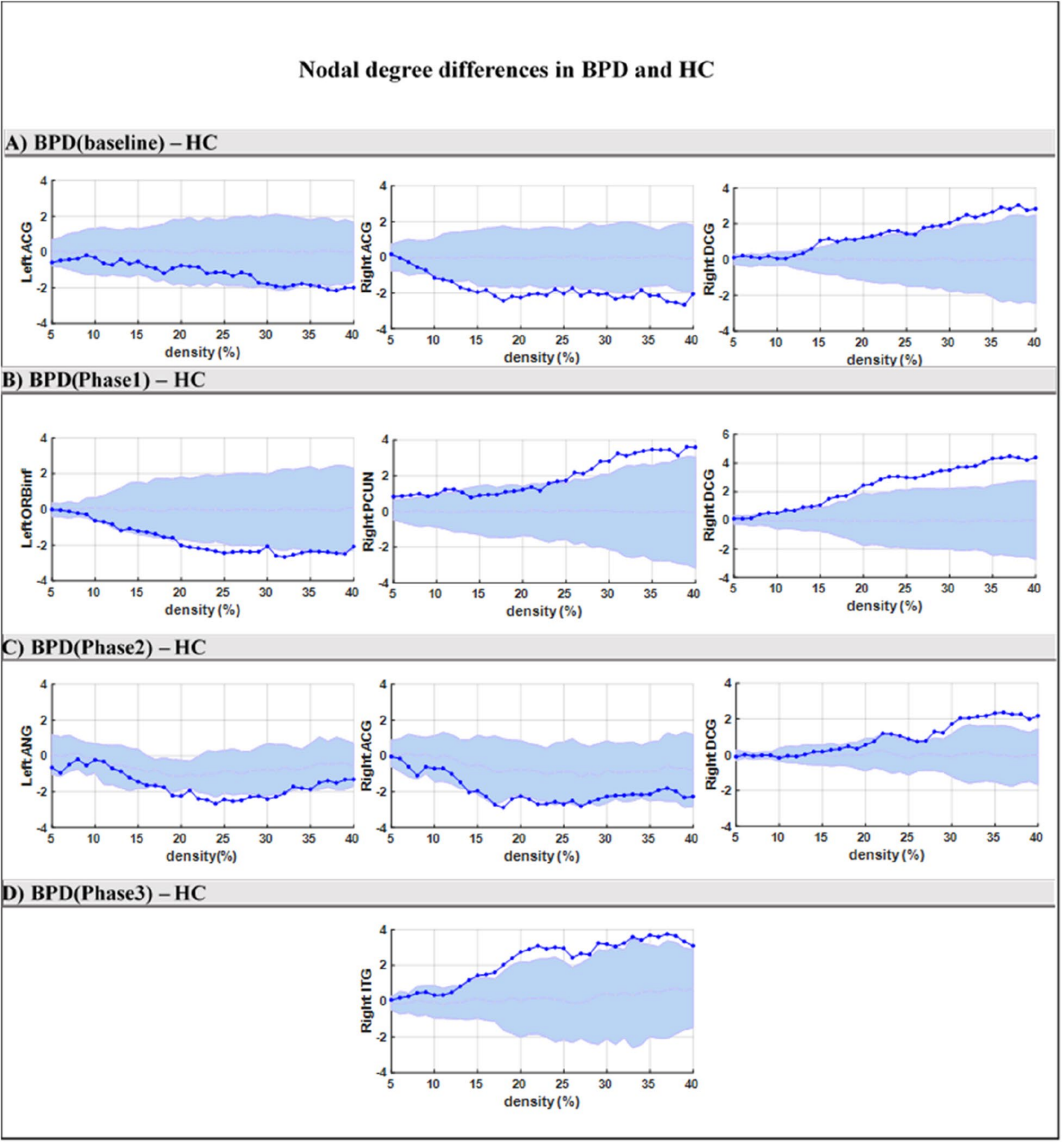
Comparison of the nodal degree values of DMN regions between the HC and BPD groups, using the non-parametric permutation test. Dark-blue points present the difference in nodal degree values between the healthy and BPD groups (BPD–HC), which lie within the confidence intervals presented by the light-blue zone. The actual difference value (dark-blue color points) is significant (< 0.05) if it falls outside the confidence intervals (light-blue zone)

We first compared the HC and BPD groups at baseline and found that the nodal degree in left and right ACG in the BPD group was *less* than in the HC group (*P* < 0.05) (Fig. 1A). Furthermore, the nodal degree in the right DCG was *greater* in the BPD (baseline) group compared to the HC group (*P* < 0.05). In other regions of the DMN, no significant difference was found between the HC and BPD (baseline) group.

#### Four months’ post-psychotherapy onset

The nodal degree was significantly greater in the BPD groups 4 months after psychotherapy (Phase1) compared to the HC group, in the right PCUN and DCG (*P* < 0.05). Also, the nodal degree in the left inferior ORB was lesser in the BPD (phase1) group compared to the HC group (*P* < 0.05) (Fig. 1B).

#### Eight months’ post-psychotherapy onset

Compared with the HC group, the BPD group, 8 months after psychotherapy (Phase2), showed a significantly greater nodal degree in the right DCG (*P* < 0.05). Furthermore, the nodal degree in the right ACG and left ANG was less in the BPD (***phase2) group compared to the HC group (*P* < 0.05) (Fig. 1C).

#### Twelve months’ post-psychotherapy onset

Comparing HC and BPD groups 12 months after the onset of psychotherapy (phase3), the nodal degree in the right ITG in the BPD group was greater than in the HC group (*P* < 0.05) (Fig. 1D). In other regions of the DMN, no significant difference was found between the HC and BPD (phase3) groups.

### DMN alternation in BPD during psychotherapy compared to the BPD in baseline

**Fig. 2:**
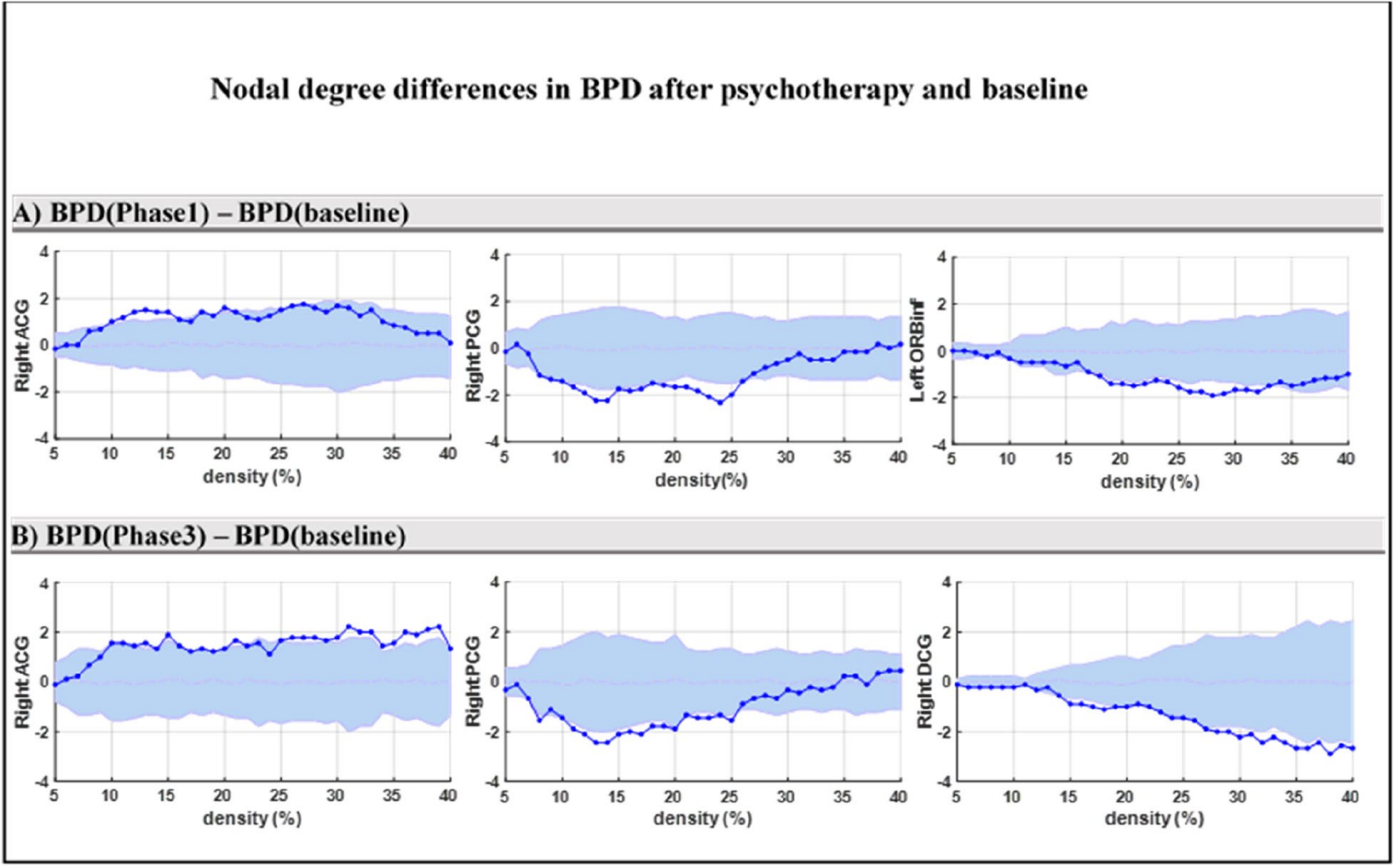
Comparison of the nodal degree values of DMN regions between the BPD in baseline and BPD groups after psychotherapy, using the non-parametric permutation test. Dark-blue points present the difference in nodal degree values between the BPD (baseline) and BPD groups after psychotherapy [BPD–BPD (baseline)], which lie within the confidence intervals presented by the light-blue zone. The actual difference value (dark-blue color points) is significant (< 0.05) if it falls outside the confidence intervals (light-blue zone)

In the DMN, the nodal degree was significantly greater in the right ACG in the BPD group 4 months after psychotherapy compared to their baseline (*P* < 0.05) (Fig. 2A). In contrast, the nodal degree was significantly less in left ORBinf and right PCG in DMN (*P* < 0.05) (Fig. 2A). In the DMN, no significant difference was found between BPD (8 months after psychotherapy) and BPD (baseline). Comparing the baseline to 12 months after psychotherapy in the BPD group, the nodal degree in right DCG and right PCG in the BPD group 12 months after psychotherapy was less than that seen at baseline (*P* < 0.05). Furthermore, the nodal degree in the right ACG was greater in the BPD group 12 months after psychotherapy compared to baseline (*P* < 0.05) (Fig. 2B).

### Alternation nodal degree of ACG, DCG in BPD pre–post-psychotherapy

**Fig. 3:**
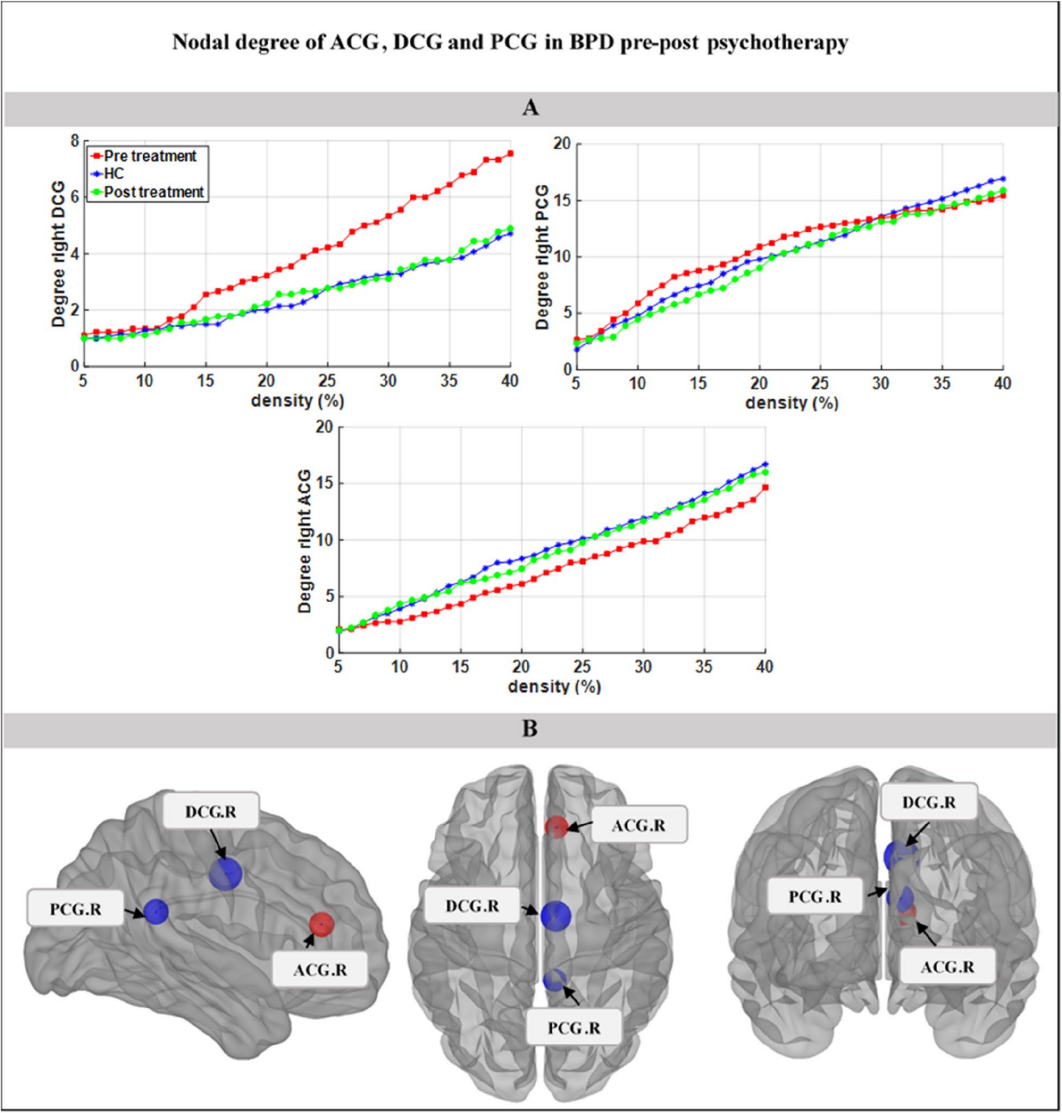
Nodal degree of ACG, DCG and PCG in BPD pre–post psychotherapy. **A** Nodal degree of right DCG and right ACC in baseline BPD (red square) or pre psychotherapy, BPD after 12 months’ psychotherapy (green circle) and HC (blue star). **B** ACG (red circle) increased nodal degree in post psychotherapy DCG and PCG (blue circle) decreased nodal degree in post psychotherapy

Figure 3A shows nodal degree of ACG, DCG, and PCG pre and post psychotherapy. Three major points emerged: (1) nodal degree of DCG and PCG after 12-month psychotherapy were significantly decreased in the BPD group. (2) In contrast, nodal degree of ACG in the BPD group after 12-month psychotherapy was significantly increased. (3) After 12-month psychotherapy, nodal degree pattern in ACG, DCG, and PCG in BPD group similar to the pattern of nodal degree these regions in the HC group. Figure 3B shows a schematic brain view of ACG, DCG, and PCG.

### Statistical analysis on clinical measurements

We found a significant positive association between the decreased nodal degree of DCG and decrease in the score of difficulty identify feeling subscale of the TAS-20 (*R* = 0.801, after FDR correction at *q* < 0.01) after psychotherapy. Results of the dependent (paired) sample *t* tests indicated that there were significant differences in the score of ASSIST, the score of BEST, and the score of TAS (difficulty identify feeling subscale) between pre- and post-psychotherapy (Table2). Mean values in post-psychotherapy for ASSIST, BEST and TAS decreased significantly (*P* value < 0.05).

**Table 2:** Differences score of ASSIST, BEST, TAS and empathy (pre–post)

Parameters	Pre(baseline) Mean ± std	Post (12 months) Mean ± std	Mean change (post–pre)	*P* value
ASSIST	13.67 ± 13.51	10 ± 10.13	− 3.67	0.021*
BEST	44.00 ± 10.05	35.89 ± 9.49	− 8.11	0.008*
TAS (difficulty identifying feeling subscale)	24.22 ± 5.14	18.89 ± 6.22	− 5.33	0.012*
TAS (difficulty describing feeling subscale)	15.89 ± 4.14	13.56 ± 3.36	− 2.33	0.264
TAS (attention)	23.00 ± 7.35	20.56 ± 3.71	− 2.44	0.458
Total TAS	63.11 ± 13.22	53 ± 8.57	− 10.11	0.087
EC (empathic concern)	16.00 ± 4.12	17.11 ± 4.80	1.11	0.481
PT (perspective talking)	14.11 ± 1.9	14.44 ± 3.88	0.33	0.784
PD (personal distress)	15.78 ± 5.54	17.33 ± 4.53	1.56	0.391
F (fantasy)	14.78 ± 4.66	15.56 ± 6.13	0.78	0.417

*P value < 0.05 is considered statistically significant

## Discussion

Our study showed that patients with BPD present with aberrant DMN connectivity compared to a matched healthy control group. However, after 1 year of psychodynamic psychotherapy, they showed neuroimaging changes [hyperconnectivity in ACC and hypo-connectivity in dACC (DCG)] and behavioral improvement in their symptoms, and decreased substance use. We also found that the decrease in dACC connectivity was associated with patient with BPD improvement in identifying their feeling. However, we did not see any changes in empathy measures after psychotherapy.

Patients with BPD demonstrated aberrant connectivity including hyperconnectivity in the dACC and hypoconnectivity in the ACC in their DMN before starting psychodynamic psychotherapy. This finding is in line with previous studies that showed abnormal connections in the DMN in patients with BPD [16, 61]. Previous studies showed structural abnormalities in GM in the DMN and frontolimbic circuit [61] and disturbed activity in the regions of the midline core and the anterior subsystem of the DMN in patients with BPD. These abnormalities are thought to reflect interpersonal emotional communication and emotion regulation difficulties in BPD [16]. More specifically, amygdala hyperactivity along with ACC hypoactivity has been proposed as the neural mechanism of emotion dysregulation in negative emotion processes in BPD [62, 63]. ACC as a part of the medial wall of the frontal lobes has been involved in emotional processing [64]. ACC abnormal activity suggests a dysfunction in frontolimibic circuitry which suggests a reduced capacity of patients with BPD to effectively activate their PFC during emotional situations leading to hyperlimbic activity and hyperarousal in these situations [65–70]. The DMN is also related to emotional self-referential processing [69–71], which is markedly affected in BPD. Accordingly, we also observed hyperconnectivity in the dACC. The dorsal portion of the ACC (dACC) is considered critical for salience detection, attention regulation and cognitive control [72–81]. dACC is a part of the default mode network and also a hub for the salience network and has some connection to PCC which is part of posterior DMN. dACC and PCC interact in focusing attention to the relevant self-related information [82, 83]. Furthermore, in emotionally charged situations, there is an interaction between attention and emotion in the dACC [84, 85]. Experimental tasks that direct attention towards emotion engage the dACC [86–88]. Greater awareness of one’s own emotional experiences is associated with greater recruitment of the dACC during emotional arousal. This finding might reflect greater attention to processing emotional information [89]. However, in patients with BPD, we found hyperconnectivity of dACC in resting state. This finding, given previous reports on patients with BPD ruminations, suggests increased attention to negative social information and enhanced self-referential processing (e.g., retrieval of negative memories of interpersonal events) during the resting state in patients with BPD.

In sum, our findings suggest aberrant connectivity in DMN and corticolimbic regions along with along with a probable changed internetwork resting state functional connectivity between salience network and DMN.

Our results, however, showed that psychotherapy may help to regulate BPD dysfunctional behavior (leading to an increase in ACC and decrease in dACC connectivity and PCC connectivity). Patients with BPD showed a reduction in symptom severity over time and a decrease in substance abuse. They also showed less difficulty in identifying their feelings and emotions. These findings were associated with a reduction in dACC hyperactivity. It is possible that psychotherapy creates a secure atmosphere to effectively process traumatic interpersonal events and emotional regulation, resulting in a DMN resting state approaching normal. Our study has some notable caveats. Due to the long course of psychotherapy, we had patients with BPD dropouts and our small sample size with a limited age range requires replication with a larger sample size. Furthermore, our exclusion criteria led to the omission of more severe patients with BPD.

## Conclusion

In BPD, there is altered connectivity within DMN and disruption in self-processing and emotion regulation. Psychotherapy may not only alleviate behavioral dysfunction but also normalizes connectivity within the DMN. This study could be a start for future studies for the investigation of the impact of the duration of psychotherapy on the pattern of DMN connectivity in personality disorders for building a time frame for psychotherapy. Evaluating DMN before and after psychotherapy might help to determine the potential target for treatment and to better identify neuroimaging results to clinical symptomatology.

## Data Availability

Data can be made available upon reasonable request.
